# The development and characterization of a 60K SNP chip for chicken

**DOI:** 10.1186/1471-2164-12-274

**Published:** 2011-05-31

**Authors:** Martien AM Groenen, Hendrik-Jan Megens, Yalda Zare, Wesley C Warren, LaDeana W Hillier, Richard PMA Crooijmans, Addie Vereijken, Ron Okimoto, William M Muir, Hans H Cheng

**Affiliations:** 1 Animal Breeding and Genomics Centre, Wageningen University, The Netherlands; 2 The Genome Institute, Washington University, School of Medicine, St. Louis, USA; 3 Hendrix Genetics Research, Technology & Services B.V., Boxmeer, The Netherlands; 4 Cobb-Vantress Inc., Siloam Springs, AR, 72761, USA; 5 Department of Animal Sciences, Purdue University, West Lafayette, IN 47907, USA; 6 USDA-ARS, Avian Disease and Oncology Laboratory, East Lansing, MI 48823, USA

## Abstract

**Background:**

In livestock species like the chicken, high throughput single nucleotide polymorphism (SNP) genotyping assays are increasingly being used for whole genome association studies and as a tool in breeding (referred to as genomic selection). To be of value in a wide variety of breeds and populations, the success rate of the SNP genotyping assay, the distribution of the SNP across the genome and the minor allele frequencies (MAF) of the SNPs used are extremely important.

**Results:**

We describe the design of a moderate density (60k) Illumina SNP BeadChip in chicken consisting of SNPs known to be segregating at high to medium minor allele frequencies (MAF) in the two major types of commercial chicken (broilers and layers). This was achieved by the identification of 352,303 SNPs with moderate to high MAF in 2 broilers and 2 layer lines using Illumina sequencing on reduced representation libraries. To further increase the utility of the chip, we also identified SNPs on sequences currently not covered by the chicken genome assembly (Gallus_gallus-2.1). This was achieved by 454 sequencing of the chicken genome at a depth of 12x and the identification of SNPs on 454-derived contigs not covered by the current chicken genome assembly. In total we added 790 SNPs that mapped to 454-derived contigs as well as 421 SNPs with a position on Chr_random of the current assembly. The SNP chip contains 57,636 SNPs of which 54,293 could be genotyped and were shown to be segregating in chicken populations. Our SNP identification procedure appeared to be highly reliable and the overall validation rate of the SNPs on the chip was 94%. We were able to map 328 SNPs derived from the 454 sequence contigs on the chicken genome. The majority of these SNPs map to chromosomes that are already represented in genome build Gallus_gallus-2.1.0. Twenty-eight SNPs were used to construct two new linkage groups most likely representing two micro-chromosomes not covered by the current genome assembly.

**Conclusions:**

The high success rate of the SNPs on the Illumina chicken 60K Beadchip emphasizes the power of Next generation sequence (NGS) technology for the SNP identification and selection step. The identification of SNPs from sequence contigs derived from NGS sequencing resulted in improved coverage of the chicken genome and the construction of two new linkage groups most likely representing two chicken micro-chromosomes.

## Background

The development of high throughput SNP genotyping assays has radically changed the genetic dissection of complex traits in human, model organisms, and agricultural species. In farm animals, for many years, such studies were focused on linkage mapping in experimental crosses with the highly variable microsatellites as the markers of choice. More recently with the development of high-density SNP chips for various farm animal species such as cattle[1], sheep [2], horses and pigs [3], these studies are increasingly being replaced by whole genome association studies. Although, the chicken was the first farm animal whose genome was completely sequenced [4] and for which large numbers of SNPs have been publically available for many years [5], the development of a high density SNP chip has not yet been described.

Nevertheless, numerous studies have used smaller scale SNP assays in chicken, in particular based on Illumina's GoldenGate assay [6-9]. These studies have clearly demonstrated the high validation rate of SNPs identified by Wong et al. [5]. However, in that study SNPs were identified by comparing the sequence of individual birds against the reference genome and no allele frequencies are available for these SNPs. Furthermore, the chicken genome assembly covers only 30 of the bird's 39 chromosomes and SNPs covering the chromosomes not represented by the current genome build are not well represented by the SNPs currently available. Lastly, while conversion rates from Wong et al. [5] have proven to be high, 50% or less are typically segregating in any single population depending on its origin and inbreeding. Our objective therefore, was to design a moderate density SNP chip in chicken consisting of SNPs known to be segregating at high to medium allele frequencies in the two major types of commercial chicken (broilers and layers) with SNPs covering all chicken chromosomes and to optimize the number of segregating SNPs for maximum utility across breeds. We used reduced representation libraries (RRL, [10]) in combination with next generation sequencing (NGS) technology for the identification of SNPs including their minor allele frequencies (MAF) in different commercial populations. To further increase the high utility of the chip we also identified SNPs on sequences currently not covered by the chicken genome assembly (Gallus_gallus-2.1). This resulted in a total number of 59,581 SNPs. We then added 790 SNPs that mapped to 454-derived contigs, 421 SNPs with a position on chr_random (Gallus_gallus-2.1) and 8 SNPs located on the mitochondrial genome for a grand total of 60,800 SNPs.

## Results

### The chicken reference genome

The SNP discovery strategy used is based on aligning short read sequences against a reference genome in order to identify variable sites [3,10]. The high sequence depth obtained by using NGS technology allows high confidence calling of SNPs and also enables estimates of MAF by counting the occurrence of the two variants. In the case of chicken, the most recent genome build is Gallus_gallus-2.1 (May 2006). This genome build covers an estimated 95% of the chicken genome, but because markers did not identify sequence in the WGS data for the 10 smallest micro-chromosomes, we believe these sequences are still not available. In order to obtain sequences from these micro-chromosomes, the Roche 454 FLX sequencing platform was used to re-sequence the genome of the same bird (UCD001) from which previous chicken genome builds were derived [4]. Since underrepresentation of the micro-chromosomes in these previous genome builds was attributed to high GC composition and cloning bias in *E. coli *[4], it was assumed that a better representation of the chicken genome could be obtained from the 454 platform as its successful use was described in human [11]. A total of 12x genome coverage of 454 sequences were assembled with Newbler (Roche 454) and non-aligned contigs to the Gallus_gallus-2.1 assembly were identified. This amounted to 72,034 supercontigs with a total length of 14.6 Mb, the majority of which (70,855) is smaller than 1kb in length. A total of 1,478 contigs with a total length of 1.17 Mb partially overlapped with sequences on chromosomes Unassigned and W. We decided to only use the sequence contigs larger than 200 bp. Setting a threshold of 200 bp these contigs were concatenated and added to the reference genome as an additional artificial chromosome, 10 Mb in size. By including these additional sequences in the SNP discovery, we anticipated identifying SNPs located on the micro-chromosomes that were not yet covered by genome build Gallus_gallus-2.1.

### SNP discovery

To obtain a large collection of SNPs and their MAF in a collection of broiler and layer breeds, we constructed 4 different reduced representation libraries (RRLs, see Material and Methods) from 4 distinct chicken populations; two broiler type chickens (B1 and B2) and a brown (BL) and white layer line (WL). Each of these was sequenced at an intended 25-40x sequence depth. Based on the size distribution of restriction fragments in the size range of 125-200 bp, we used the restriction enzyme *Alu*I for the construction of the libraries. We aimed for a genome coverage of around 2% and a sequence depth of 25x. Initially we isolated fragments in the size range from 150-250 bp (BL and WL), which resulted in genome coverage of around 1.4% and a sequence depth of 45x. For the broiler lines (B1 and B2) we therefore increased the size range of the fragments to be sequenced to 125-200 bps, which resulted in a genome coverage of 2.9% and sequence depth of 37x for B1 and a genome coverage of 4.6% and sequence depth of 18x for B2. In total, we generated 121,528,957 quality approved 36 bp paired end sequences and after further filtering the sequences (see Material and Methods), 88,183,348 sequences were used to align against the chicken reference genome described above and for subsequent SNP identification. SNP identification was performed with MAQ 0.6.6. [12] essentially as described previously [3].

**Figure 1:**
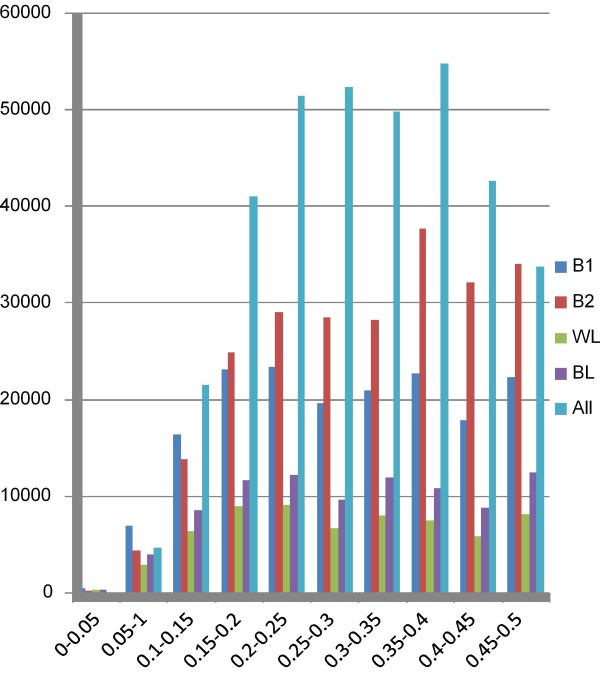
**Distribution of estimated MAFs**. The MAF for all 352,303 SNPs identified by Illumina sequencing was estimated based on the read counts for the two alleles. MAFs were calculated individually for all 4 populations used in the SNP discovery and as an average over all 4 populations combined (All).

SNP identification was done separately for each of the 4 lines (Table 1) after which the results were combined. After applying stringent filtering criteria, in particular that both variants should have been observed at least three times each, a total of 352,303 SNPs were identified. About half (151,280) of these SNPs were detected in at least two of the breeds and 55,190 were already present in dbSNP release 122. The number of SNPs identified in the 4 different lines is shown in Table 1. The average MAF of the SNPs identified is 0.30 and the MAF spectrum of the SNPs shows a rather flat distribution with the majority of the SNPs showing high MAFs (Figure 1).

**Table 1:** SNPs identified in the 4 populations used

line	B1	B2	BL	WL	Percentage of the genome covered
B1	173,830	93,156	49,607	29,802	2.85

B2		233,055	48,342	31,904	4.64

BL			90,405	23,941	1.47

WL				64,072	1.24

The number on the diagonal is the number of SNPs identified in that particular population The other numbers show the number of SNPs that are in common between any pair of populations

Recently over 7,451,250 additional SNPs were identified by Rubin et al., [13] located on chromosomes 1-28 and 32. Comparison of the 326,895 SNPs located on the chromosomes covered by the submission of Rubin et al. (2010) showed that around 50% of our SNPs (168,281) were common to the two sets. A comparison of the 2.5 million SNPs identified by Wong et al., [5], excluding indels and SNPs on the Z chromosomes, with the SNPs identified by Rubin et al. [13] shows that 62% of the SNPs identified by Wong et al [5] are present in the Rubin et al [13] data set.

### SNP selection and chip design

To maximize the number of SNPs on the chicken iSelect chip to be designed, we decided to only include Infinium type II SNPs (A/C; A/G; T/C; T/G) because these SNPs require only a single bead type on the chip [14]. The number of Infinium type II SNPs within the total number of 352,303 SNPs identified was 306,706. A second strict criterion used to increase the success rate of the SNPs on the chip was that the SNPs had to have an Illumina design score of at least 0.6. The Illumina design score (scale 0-1) reflects the ability to design a successful assay. This further reduced the number of SNPs available for the final selection to 196,384. Similar criteria were used to select SNPs from dbSNP for the final SNP selection resulting in 1,838,048 SNPs. Because both sources, the Illumina SNP set and the dbSNP set, had 44,149 SNPs in common, the total number of SNPs used for the selection of the SNPs for the Illumina bead chip was 1,990,028.

**Figure 2:**
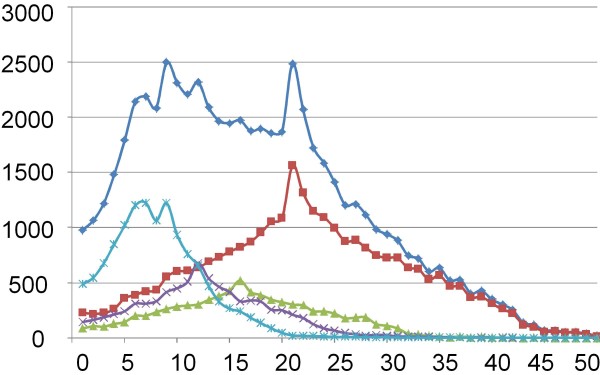
**Distribution of spacing of SNPs selected for the Illumina 60k BeadChip**. Spacing between the SNPs was calculated for different chromosomes: All (dark blue), 1-5 (red), 6-9 (green), 10-14 (purple) and 15-28 (light blue). The spacing (x-axis) is shown in Kb.

From this final list we selected 60,800 SNPs to be included on the Illumina iSelect beadchip. These 60,800 SNPs were selected based on a priority score as described in the Material and Methods. The combination of the different criteria (validation, Illumina design score, MAF information in broilers and layers) resulted in a total of 25 priority classes. For example, SNPs that had previously been validated in an Illumina genotyping assay and that had a high MAF were given the highest priority during the selection process, whereas SNPs from dbSNP that had not previously been used and therefore no MAF was available were given the lowest priority. The final step of the selection process was to scroll along the individual chromosomes and to select SNPs with the highest priority value at regular distances along the chromosome. The spacing of the SNPs on the different chromosomes was varied based on the size of the chromosome (see Additional file 1). This resulted in a total number of 59,581 SNPs. We then added 790 SNPs that mapped to 454-derived contigs, 421 SNPs with a position on chr_random (Gallus_gallus-2.1) and 8 SNPs located on the mitochondrial genome (Table 2). The final distribution of the spacing between the SNPs selected for the 60K iSelect chip is shown in Figure 2.

**Table 2:** Origin of SNPs selected for the Illumina 60k BeadChip

Selected SNPs on the chip	Number
Total number of SNPs selected	60,800

dbSNP all	41,895

SNP in dbSNP and previously validated	17,545

SNP in dbSNP not validated	24,350

RRL all	26,551

Detected in RRL not in dbSNP	18,758

Detected in RRL and present in dbSNP	7,793

SNPs unmapped	1,211

### Performance of the chicken 60K iSelect beadchip

Of the 60,800 SNPs submitted to Illumina to be included on the iSelect BeadChip, a total of 57,636 were eventually retained for the analysis. To evaluate the performance of the Illumina chicken iSelect BeadChip, the 57,636 markers that passed the assay design tool informatics screen was used to genotype a range of individuals representing a wide variety of breeds. These included several commercial broiler and layer lines as well as Dutch fancy breeds and two different populations of the red jungle fowl. Based on assay design and performance all 60,800 were grouped in one of the following four classes: (1) "failed" or those SNPs that did not pass the Illumina assay design thresholds, (2) "excluded" or SNPs that were excluded after detailed evaluation of the clustering results; the majority of SNPs within this group either could not be unequivocally clustered in the three genotype classes or consisted of only heterozygotes, (3) "monomorphic" or SNPs for which only a single allele was observed, and most likely representing false positive SNPs from the original SNP discovery process, or (4) "polymorphic", SNPs for which both alleles were observed in at least one of the breeds examined.

To evaluate the SNP discovery process, SNPs were also classified based on the procedure used for SNP discovery (low coverage Sanger sequencing [5] or Illumina sequencing of RRLs and whether SNPs had previously been used in SNP genotyping assays. The distribution of the 4 performance classes for each of these groups of SNPs is shown in Table 3.

**Table 3:** Performance and validation of SNPs based on origin of discovery

Group	Failed design		Excluded from analysis		Monomorphic		Polymorphic	
	
	number	%	number	%	number	%	number	%
All SNPs	3164	5.0	1116	1.9	2227	3.9	54293	94.2

dbSNP confirmed	893	5.1	64	0.4	27	0.2	16629	99.5

dbSNP random	886	5.1	764	4.6	1581	9.6	14087	85.7

Illumina sequencing	1431	5.3	285	1.1	618	2.4	24356	96.4

The percentage shown is relative to the group of SNPs described in a specific row.

### Mapping unassigned SNPs

To include regions not well covered by genome build Gallus_gallus-2.1.0, 1211 SNPs were included that were derived from Chr_random or from 454-based sequence contigs whose sequences were not included in this genome build. To be able to map these SNPs on the chicken genome, we genotyped two families of the Wageningen mapping population [15] with the chicken iSelect Beadchip. These families were recently used to develop improved high resolution SNP based linkage maps of the chicken genome [7,16]. Although the number of offspring genotyped was relatively small (92), limiting the mapping resolution, the total number of SNPs used (57,636) guarantees a high power to observe linkage between multiple markers and thus, results in a high power to map unmapped SNPs. Of the 1211 unmapped SNPs, 431 could be assigned to a chromosomal location or to a new linkage group (Additional file 2). Seventy-seven of the 421 SNPs from Chr_random could be positioned on a chromosome, and all of these, except the SNPs from Chr25_random, mapped to specific locations on the expected chromosomes. The SNPs that previously were assigned to Chr25_random, all map to the linkage group for chromosome 24.

**Figure 3:**
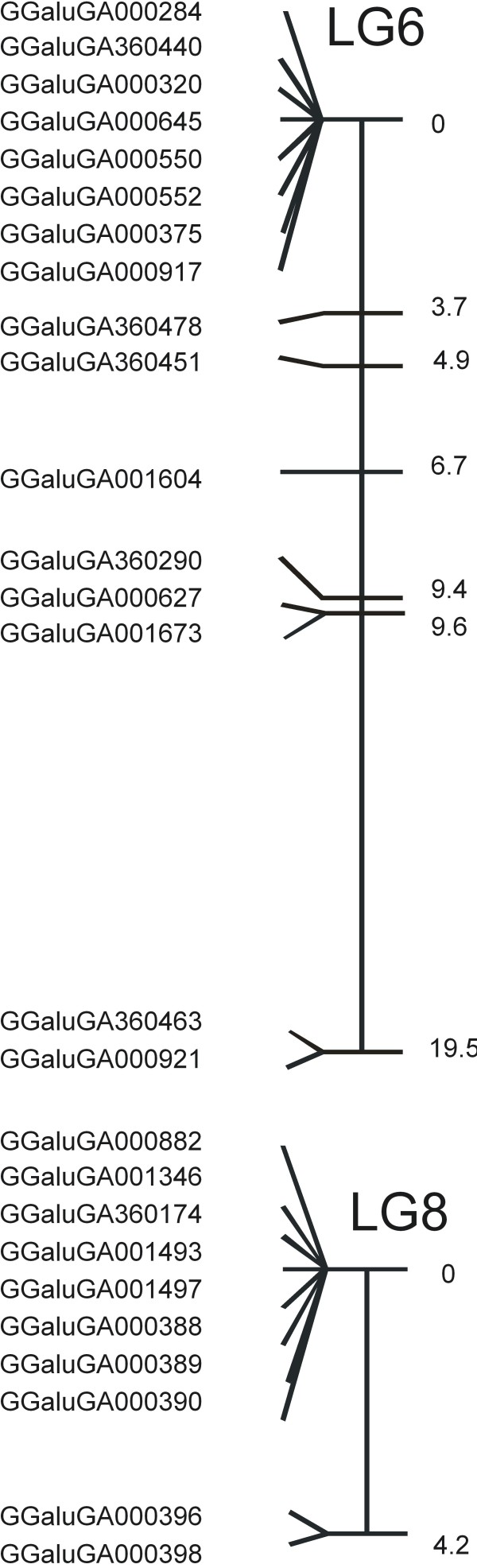
**Comprehensive linkage maps of linkage groups LG6 and LG8**. Marker spacing is shown in cM and mapping function used within Crimap is based on Kosambi.

The majority of the 328 SNPs derived from the 454 sequence contigs, map to chromosomes that were already represented in genome build Gallus_gallus-2.1.0 and only 28 SNPs map to two new linkage groups (LG6 and LG8; Figure 3). A third new linkage group (LG7) was obtained and consists of 12 SNPs previously located on ChrE64_random, 12 SNPs from ChrW_random, and 2 SNPs derived from 454 sequence contigs.

To further evaluate the new linkage groups, we aligned the sequences of the 454 contigs with the SNPs that map to these linkage groups to the human genome. The 454 contigs of 8 of the SNPs located on linkage groups LG6 and LG8 aligned to the human genome. Two contigs aligned to sequences on human chromosome 19q13, three to human 17q, and three to human 12q13.

## Discussion

The Illumina chicken 60K SNP BeadChip described in this paper was shown to contain a large percentage (>94%) of SNPs that are segregating in a variety of chicken breeds and that can reliably be genotyped. Our approach to identify a large number of new SNPs and to determine their MAF was shown to be a very efficient, cost effective approach in the design of the chip. Although, previous results [6,15] had already shown that the conversion rate of a large proportion of the SNPs identified by Wong et al. [5] was very high, the conversion rate of the SNPs identified by our approach surpassed those identified by Wong et al. [5]. Of the new SNPs identified in this study, more than 96% could be validated, whereas only 86% of the SNPs present in dbSNP could be validated. To some extent this likely is due to the selection of SNPs with a high MAF using our Illumina sequencing approach. Nevertheless, it also reflects the power of the high sequence depth (18-45x) that was obtained using our RRL-Illumina sequencing approach as compared to the low sequence depth (2-3x) generally obtained using classical Sanger sequencing [5].

This high sequence depth allowed stringent filtering criteria reducing the number of false positive SNPs even though the percentage of sequencing errors in the Illumina sequence is much higher compared to sequences obtained by Sanger sequencing. Likewise, the ability to estimate MAF due to the large number of haplotypes sequenced, enabled the selection of common SNPs and the exclusion of rare SNPs. This is not possible in cases where only two or three haplotypes are sequenced. Our results clearly show that with the current low sequencing costs of massive parallel sequencing techniques, a SNP identification step is recommended even in situations where a large number of SNPs is already available. In particular, the additional information of the estimated MAF strongly improves the usefulness of the final product (i.e., genotyping assay). Finally, as expected, the majority of the SNPs that earlier had been used in other genotyping assays and that were included on the chip were validated in our study (99.5%).

A second important criterion for a whole genome genotyping assay is the uniform distribution of the SNPs across the genome, as this greatly facilitates finding associations between markers and phenotypes. In our chip design, therefore, much emphasis was placed on the location of the SNPs during the SNP selection phase of the project. Important in this respect is the availability of a large collection of SNPs to choose from during the design of the chip. As a rule of thumb, the number of SNPs should be at least 10-fold higher than the targeted number for the final chip. This allows the inclusion of further criteria in addition to the spacing criterion. Because of the much higher recombination rate of the micro-chromosomes, we targeted a 4-fold higher SNP density on the micro-chromosomes. Based on LD analysis of a variety of chicken populations from previous studies [8] and results obtained with the 60k chip, we would advise an even larger difference (6-8 fold) in SNP density on the micro-chromosomes-compared to the macro-chromosomes for future SNP chip design in chicken and other birds.

Finally, the third important criterion for a whole genome SNP assay is the complete coverage of the genome. Although this might seem trivial, for chicken (and probably birds in general) this offers additional difficulties and challenges. For all the birds whose genome has been sequenced so far (chicken (4], zebra finch [17], and turkey [18]), it has proven to be extremely difficult to obtain a good uniform coverage of the genome. Although the reason is still not known, sequences representing the smallest micro-chromosomes are highly underrepresented in the available genome assemblies. We aimed to solve this problem by sequencing the chicken genome at a depth of >12x using a second generation sequencing approach (Roche 454). We obtained >10 Mb of sequences that were not covered by assembly Gallus_gallus-2.1, which is based completely on traditional Sanger sequences. However, of the 790 SNPs that are derived from these sequences, only 28 could be assigned to two new linkage groups, likely representing two of the micro-chromosomes still missing from the current assembly. Surprisingly, the majority of the SNPs derived from these sequences map to chromosomes already represented in the current genome build. This highlights that even the better assembled chromosomes within assembly Gallus_gallus-2.1 contain underrepresented regions. Moreover, the underrepresentation of the micro-chromosomes in genome assemblies does not seem to stem solely from a cloning bias but rather appears to point to a feature of the micro-chromosomes that affects 454-based sequencing as well. It was suggested [4] that the higher GC content or (chromosome specific) repeats might contribute to the absence of the micro-chromosomes from the assembly. Interestingly, sequences from the smallest micro-chromosomes are also under represented in Illumina based sequences from turkey [18].

In total, 328 SNPs derived from the 454 sequence contigs were assigned a chromosomal location, allowing further improvement of future genome builds for chicken. The same applies to the 77 SNPs from Chr_random that could be assigned to a specific location on the linkage map. The new SNPs do increase the fraction of the genome that is covered by the 60K chip and they allow the analysis of two putative additional chicken chromosomes. Previously, it was shown [4] that specific regions e.g. regions syntenic to human chromosomes 19 were underrepresented in the chicken genome assembly. Mapping two contigs of the new linkage groups to this region on human chromosome 19 is strong support that indeed these syntenic regions are located on micro-chromosomes in birds. The improvement of the coverage of future (more dense) SNP chips, in particular the coverage of the missing micro-chromosomes, clearly remains a huge challenge for birds. Improving our understanding of which syntenic regions in non-bird species may map to bird micro-chromosomes is therefore important for ultimately understanding why these sequences are so difficult to obtain in birds. The involvement of cloning (Sanger sequencing) or PCR (454, Illumina) steps in combination with specific sequence features of the micro-chromosomes likely underlie this difficulty. Therefore, new sequencing technologies based on single molecule sequencing might offer further opportunities to eventually obtain a genome build and SNP chip that in actuality covers the vast majority of the chicken genome.

## Conclusions

The high success rate of the SNPs on the Illumina chicken 60K Beadchip emphasizes the power of Next generation sequence (NGS) technology for the SNP identification and selection step. The identification of SNPs from sequence contigs derived from NGS sequencing resulted in improved coverage of the chicken genome and the construction of two new linkage groups most likely representing two chicken micro-chromosomes.

## Methods

### Animals and DNA samples

DNA samples were obtained from 4 commercial breeding lines, two meat-type chicken (broilers, lines B1 and B2) and two egg-type chicken (brown and white type layers, lines BL and WL, respectively) breeding lines. For each of these lines, whole blood from 25 individual animals was pooled [19] and high quality high molecular weight DNA exacted using the Puregene system (Gentra, USA).

### Chicken Genome sequencing

DNA from the female red jungle fowl RJF #256 from the inbred line UCD001, previously used to build assembly Gallus_gallus-2.1, was randomly sheared and used to prepare three different libraries for sequencing on the Roche 454 Titanium and FLX sequencing platforms The sequences used for the assembly of additional contigs not represented in assembly Gallus_gallus-2., included 10X of Titanium fragments, 1.7X of 3 kb insert paired ends using the 454 FLX platform, and 1.2X of 20 kb paired end Titanium reads. These additional sequences are available at http://genome.wustl.edu/genomes/view/gallus_gallus/.

### Construction of reduced representation libraries and sequencing

In order to reduce complexity, we generated four reduced representation libraries (RRLs) for the four chicken lines. For each pool, 25 µg of DNA was digested with *Alu*I (Fermentas GmbH, St Leon-Rot, Germany) following the manufacturer's recommendations. The restriction enzyme *AluI *was chosen empirically, after evaluation of the percentage of the genome represented by fragments in the size range of 150-200 bp. Upon completion of each digestion, fragments were fractionated with a 10% non-denaturing polyacrylamide Criterion TBE gel (Biorad, Veenendaal, Netherlands) at 100 V for 190 min. DNA fractions were stained by immersing a gel for 15 min in a TBE 1x solution containing ethidium bromide. Fragments in the size range of 150-200 bp (lines BL and WL) and 125-200 bp (lines B1 and B2) were excised from the gel as described previously by Ramos et al[3]. A Gel Doc XR (BioRad) was used to estimate the fraction of the genome covered by the libraries. For library preparation, the Genomic DNA Sample Prep Kit (Illumina) was used according to the manufacturers' instructions. All sequencing was done on an Illumina 1G sequence analyzer resulting in paired end sequences of 36 bp each.

### SNP discovery

Before aligning the Illumina reads against the chicken reference genome (Gallus_gallus-2.1.0; Hillier et al., 2004), reads were filtered according to the quality values and presence of the *Alu*I restriction motif at the start of the reads. Reads were removed from the analysis if the average quality score was below 12 and if the first two bases were not "CT." Next, sequence reads that were likely to be derived from repetitive sequences in the genome were also removed. These included reads containing homopolymers of >17 contiguous bases (≥0.5x read length of 36 nucleotides) or reads mapped to sites that were overrepresented (observed more than five times the estimated average sequence depth). Sequences were aligned against build Gallus_gallus-2.1.0 of the chicken genome supplemented with additional contigs obtained by 454 sequencing. Initial SNP detection was performed using MAQ version 0.6.6 using the default settings (Li et al., 2008). For SNP discovery, only reads that aligned to a single unique location of the genome were considered. Further criteria used to exclude less reliable SNPs from the dataset included a minimal map quality for the read of 10, minimal consensus quality of 10 and a minimal map quality of the best mapping read of 10 for each predicted SNP position. Moreover, we also required the minor allele at each SNP be represented by at least three reads. To minimize the number of false positives due to paralogues sequences, a maximum read depth of 4 times the average read depth was used. Finally, SNP MAFs were estimated by directly counting the number of reads for each allele. All de novo identified SNPs have been submitted to dbSNP (accession numbers from ss316921455 to ss317554408 and from ss325994936 to ss325995061; see Additional file 1).

### Selection of the final SNP list

All chicken SNPs available in dbSNP release 122 were downloaded and submitted to Illumina for design score calculation, which was performed with Illumina's Assay Design Tool for Infinium. We only considered SNPs with a design score above 0.6 in the design of the chip. Furthermore only type II Infinium SNPs were included because these SNPs require only a single bead type on the chip. In addition to the Illumina design score and the type of Infinium assay, we also considered other parameters, such as the estimated MAF, spacing of the SNPs along each chromosome, genome wide coverage (number of SNPs selected for each chromosome), the presence of other SNPs within 10 bp of each target SNP and available information concerning the conversion rate of the SNP (i.e., had the SNP been successfully assayed before). Information for these parameters was collected for all available SNPs and was used to assign SNPs to 25 different priority classes [3,9]. Using a custom perl script, SNPs were selected based on priority class and position on the chromosomes. Because in chicken recombination is inversely correlated with chromosome size (Hillier et al., 2004; Groenen et al., 2009), SNPs were spaced to give a similar recombination rates regardless of chromosome with spacing ranging between SNPs from 20,000 bp for the macro-chromosomes to 4,000 bp for the micro-chromosomes (Additional file 3).

### SNP genotyping and linkage mapping

Samples were genotyped according to the procedures described by Illumina. Markers were evaluated for signal intensity, robust cluster formation and cluster separation. Genotyping and quality control was done using the standard protocol for Infinium iSelect Beadchips implementing BeadStudio Genotyping v3.0.19.0. (http://www.illumina.com/Documents/products/technotes/technote_infinium_genotyping_data_analysis.pdf). Linkage mapping was performed with a modified version of CRI-MAP [20] capable to handle large data sets and was provided by Drs. Liu and Grosz, Monsanto Company, St. Louis, MO, USA. Mendelian inheritance errors were identified using the option 'prepare' and removed from the data. Initially, SNPs were assembled in separate chromosome-specific files based on previous linkage information [7,16] and the chicken genome sequence [4]. Subsequently, all unmapped SNPs were compared to all other SNPs using the CRI-MAP option 'twopoint'. Loci that did not show linkage to multiple markers within the same chromosome were combined in a separate file with other unassigned markers. Finally all unassigned markers were compared against each other using the CRI-MAP option 'twopoint'. New linkage groups were only constructed in case individual markers were linked with a LOD score higher than 4. Construction of the new linkage groups was as described previously [16].

## Authors' contributions

MAMG analysed the data, designed the chip and drafted the manuscript, HJM and YZ helped with the analysis, RPMAC constructed the RRLs, WCW and LWH performed the 454 sequencing and genome assembly, AV, RO, WMM and HHC helped in the selection of the SNPs for the chip. All authors were involved in improving the manuscript. The final version of the manuscript was approved by all the authors.

## Supplementary Material

Additional file 1 **A tab delimited flat text file with the dbSNP accession numbers for the SNPs identified using Illumina sequencing of RRLs**. Click here for file

Additional file 2 **An Excel file with the chromosomal positions of the SNPs positioned on the chicken linkage map**. Click here for file

Additional file 3 **An Excel file with for each individual chromosome, the number and average spacing of the SNPs present on the Illumina Beadchip**. Click here for file
